# Integrating genome-wide association study with transcriptomic data to predict candidate genes influencing *Brassica napus* root and biomass-related traits under low phosphorus conditions

**DOI:** 10.1186/s13068-023-02403-2

**Published:** 2023-10-04

**Authors:** Nazir Ahmad, Sani Ibrahim, Lieqiong Kuang, Tian Ze, Xinfa Wang, Hanzhong Wang, Xiaoling Dun

**Affiliations:** 1 Oil Crops Research Institute of the Chinese Academy of Agricultural Sciences/Key Laboratory of Biology and Genetic Improvement of Oil Crops, Ministry of Agriculture and Rural Affairs, Wuhan, 430062 China; 2 Department of Plant Biology, Faculty of Life Sciences, College of Physical and Pharmaceutical Sciences, Bayero University, P.M.B. 3011, Kano, 700006 Nigeria; 3 Hubei Hongshan Laboratory, Wuhan, 430062 China

**Keywords:** Rapeseed, GWAS, Biomass traits, QTL, Phosphorus, PUE

## Abstract

**Background:**

Rapeseed (*Brassica napus* L.) is an essential source of edible oil and livestock feed, as well as a promising source of biofuel. Breeding crops with an ideal root system architecture (RSA) for high phosphorus use efficiency (PUE) is an effective way to reduce the use of phosphate fertilizers. However, the genetic mechanisms that underpin PUE in rapeseed remain elusive. To address this, we conducted a genome-wide association study (GWAS) in 327 rapeseed accessions to elucidate the genetic variability of 13 root and biomass traits under low phosphorus (LP; 0.01 mM P +). Furthermore, RNA-sequencing was performed in root among high/low phosphorus efficient groups (HP1/LP1) and high/low phosphorus stress tolerance groups (HP2/LP2) at two-time points under control and P-stress conditions.

**Results:**

Significant variations were observed in all measured traits, with heritabilities ranging from 0.47 to 0.72, and significant correlations were found between most of the traits. There were 39 significant trait–SNP associations and 31 suggestive associations, which integrated into 11 valid quantitative trait loci (QTL) clusters, explaining 4.24–24.43% of the phenotypic variance observed. In total, RNA-seq identified 692, 1076, 648, and 934 differentially expressed genes (DEGs) specific to HP1/LP1 and HP2/LP2 under P-stress and control conditions, respectively, while 761 and 860 DEGs common for HP1/LP1 and HP2/LP2 under both conditions. An integrated approach of GWAS, weighted co-expression network, and differential expression analysis identified 12 genes associated with root growth and development under LP stress. In this study, six genes (*BnaA04g23490D, BnaA09g08440D, BnaA09g04320D, BnaA09g04350D, BnaA09g04930D, BnaA09g09290D*) that showed differential expression were identified as promising candidate genes for the target traits.

**Conclusion:**

11 QTL clusters and 12 candidate genes associated with root and development under LP stress were identified in this study. Our study's phenotypic and genetic information may be exploited for genetic improvement of root traits to increase PUE in rapeseed.

**Supplementary Information:**

The online version contains supplementary material available at 10.1186/s13068-023-02403-2.

## Background

The macronutrient phosphorus (P) is one of the most essential micronutrients that plants require for several basic physiological functions, including energy production, glycolysis, nucleic acid synthesis, enzyme activation/inactivation, redox reactions, carbohydrate metabolism, and nitrogen fixation [1, 2]. Low availability and inadequate P supply are the major constraints for crop production [3, 4]. The cropland in many regions of the world is deficient in phosphorus, especially in developing countries [5]. The excessive application of P fertilizers and P-deposition into rivers have caused a low use efficiency of P fertilizers, adversely affecting the environment and the ecology [6]. As a result, it is essential to develop genotypes with high phosphorus use efficiency (PUE) that may perform well in P-deficient conditions [7].

The physiological properties of the plant's root system heavily influence how quickly crop plants absorb nutrients and water [8]. As a result of P-stress, plants can stimulate root hair growth, increase lateral root growth, reduce primary root growth, increase root surface area, increase root volume, shallower root growth angle, and improve root biomass [9, 10]. P deprivation induces the formation of a highly branching root system at the expense of the primary root in *Arabidopsis*, as shown by the increased formation and appearance of lateral roots and root hairs [11, 12]. Low P availability has been found to affect the growth of primary root, root angle, branching of roots, lateral roots, root hair augmentation, and cluster root formation in crops such as maize and rice [13, 14]. Moreover, it has been observed that genetic improvement of root morphological traits affects crop yield [15, 16]. Rice yield has been shown to increase under phosphorus stress when *PSTOL*, a gene associated with phosphorus stress tolerance, was evaluated in overexpressed lines [17]. The gene *ACP1* encoding acid phosphatase has been reported to affect root hairs and seed yield under P-deficiency in soybean [18]. Early seedling variations correlate well with the high P uptake. A deeper understanding of the biomass and root traits like root length, root fresh weight, root biomass, and shoot biomass can throw some light on dissecting mechanisms underlying PUE. Hence, exploring the variations for seedlings and their initial biomass accumulation traits under limiting P conditions would provide a better scope for improving elite lines for PUE [19].

Rapeseed (Canola, *Brassica napus* L.) is a worldwide important source of oil, accounting for 20% of global vegetable oil for human consumption, industrial oils, biodiesel, lubricant, and fodder for animal feeds [20, 21]. In addition, rapeseed is a phosphorus deficiency-sensitive crop as well [22, 23]. Phosphorus deficiency seriously affects rapeseed yield, quality, and resistance to various environmental stresses. Coarse root length in rapeseed improves soil exploration and phosphorus uptake, which boosts seed production [24]. Hence, addressing the genetic relationship between root architectural traits and PUE can provide basic information for improving rapeseed's P acquisition and yield.

GWAS is an efficient approach to dissecting complex traits' genetic basis, including root architectural traits using naturally occurring genetic diversity [25]. The GWAS technique has recently been widely used for root and biomass traits in many crops, including rice, maize, wheat, soybean, sesame, and rapeseed [5, 18, 26–30]. RNA-sequencing (RNA-seq) can be used to describe or identify genes and acquire accurate transcript levels. In addition, it is also a valuable tool for dissecting gene regulation networks by identifying differentially expressed genes (DEGs). The weighted gene co-expression network analysis (WGCNA), one of the most popular methods for discovering hub factors controlling traits, allows for discovering core gene networks by analyzing gene expression patterns from RNA-seq data [31, 32]. Finding candidate genes for complex traits can be accomplished quickly and efficiently by integrating GWAS, transcriptome sequencing, and WGCNA. Two hub genes (*GRMZM2G075104* and *GRMZM2G333183*) were identified in response to salt stress by combining GWAS and WGCNA [33]. In another study, three critical genes (*BnaA09g06990D, BnaC04g39510D,* and *BnaC08g26920D*) were detected by combing GWAS and WGCNA in response to lodging-related traits in rapeseed [34]. With the help of GWAS, differential expression analysis, and WGCNA, four and eight crucial candidate genes related to root growth in rapeseed were identified during the persistent and specific stages, respectively [29].

The phenotyping of root architectural traits is challenging due to the hidden feature of roots. Different root phenotyping platforms have recently been utilized to assess root traits, including sand, germination paper, hyperspectral imaging, and hydroponic-based culture [35]. Hydroponic culture combined with digital imaging can rapidly and precisely detect a variety of root traits in large populations with different aspects of root development compared with sand culture and germination paper techniques [36]. It has been used for studying root architecture differences in various crops, including rice, wheat, maize, soybeans, and rapeseed [28, 29, 37–40].

An association panel of 327 *B. napus* cultivars that were genotyped using the *50K Brassica Infinium SNP array,* to investigate 13 root and biomass traits under control (CK) and low phosphorus (LP) treatments in hydroponic culture [28]. RNA-seq was conducted in two growth stages of high and low phosphorus efficient/tolerance groups to examine the expression levels of candidate genes. The current work attempted to identify significant SNPs linked to root and biomass traits related to PUE and used GWAS and RNA-seq techniques to determine potential candidate genes that regulate root response to LP stress. This study will provide knowledge on the phenotypic variability in rapeseed root architectural traits for mapping important QTL and molecular markers. In addition, the integrated GWAS, RNA-seq, and WGCNA will give us an overview of low phosphorus mechanisms at the seedling stage of rapeseed, further expanding our understanding of rapeseed adaptation to P deficiency, providing a better understanding of the molecular relationships involved in the definition of the phenotypes of interest and thus supplying research support for the development of appropriate genomic-based strategies for rapeseed breeding.

## Results

### Performance of eight lines under P-concentration gradients

Two independent experiments were conducted with eight lines, H1, H2, H3, H4, H5, H6, H7, and H8, chosen at random from the *B. napus* association panel and cultivated hydroponically with eight P levels ranging from P1 to P8 at a concentration of 1, 0.1, 0.05, 0.025, 0.01, 0.007, 0.005 and 0.003 mM P + , respectively. To determine the effect of different levels of phosphorus deficiency on rapeseed growth, the eight lines’ shoot fresh weight (SFW) was examined (Additional file 1: Fig. S1a). As shown (Additional file 1: Fig. S1a–b), SFW decreased with decreasing treatment concentrations, demonstrating that P stress profoundly affects plant growth. The SFW of all eight lines declined significantly at 0.01 mM P^+^ (P5) compared with one mM P^+^ control, with a ratio of less than 50% (Additional file 1: Fig. S1b). Furthermore, there were significant differences between the genotypes regarding their SFW ratio, with values ranging from 14.3 to 40.9% (Additional file 1: Fig. S1b), suggesting that phosphorus uptake efficiency differs significantly among genotypes of *B. napus*.

### Phenotypic variations of root and biomass traits under LP stress in the association population

Thirteen root and biomass-related traits (Table 1) were investigated in 327 *B. napus* association mapping panel under control (1 mM P^+^) and low phosphorus stress (0.01 mM P^+^) treatments. Under control and LP conditions, the coefficient variation of the 13 traits ranged from 14.5 to 45.6% and 10.0–32.0%, respectively. For all the studied traits, moderate-to-high heritability of 0.47–0.72 was observed in both conditions (Table 2). Furthermore, our findings demonstrated that phosphorus stress had a detrimental effect on normal root and shoot growth, resulting in a decrease in shoot fresh weight (SFW) by 131.8%, shoot dry weight (SDW) by 76.8%, total fresh weight (TFW) by 89.2%, and total dry weight (TDW) by 60.7% (Table 2). Some of the studied traits were increased significantly under LP stress; primary root length (PRL) by 8.5%, root fresh weight (RFW) by 13.4%, total root length (TRL) by 14.9%, total root surface area (TSA) by 17.1%, total root volume (TRV) by 15.4%, total root numbers (TRN) by 10.3%, root dry weight (RDW) by 16.1%, the root–shoot ratio in fresh weight (RSRF) by 62.9% and root–shoot ratio in dry weight (RSRD) by 53.8% (Table 2).

**Table 1:** Description of the 13 examined traits

Classification	Trait description	Abbreviations	Units
Root morphological traits (MT)	Primary root length	PRL	cm
	Total root length	TRL	cm
	Total root surface area	TSA	cm^2^
	Total root volume	TRV	cm^3^
	Total number of roots	TNR	Number
Biomass-related traits (BM)	Shoot fresh weight	SFW	g
	Root fresh weight	RFW	g
	Shoot dry weight	SDW	g
	Root dry weight	RDW	g
	Total fresh weight	TFW	g
	Total dry weight	TDW	g
	Fresh root–shoot ratio	RSRF	RFW/SFW
	Dry root–shoot ratio	RSRD	RDW/SDW

**Table 2:** Descriptive statistics for investigated traits under control and low phosphorus treatment in association panel

Trait	Control					LP					Control × *P*-stress	*N*-stress impact (%)
	Min	Max	Mean	CV (%)	*h*^2^	Min	Max	Mean	CV (%)	*h*^2^		
PRL (cm)	12.2	34.3	24.3	14.5	0.62	18.8	34.6	26.5	10.0	0.49	**	8.5
TRL (cm)	434.3	1435.7	843.8	20.7	0.66	500	1834	991	18.4	0.56	**	14.9
TSA (cm^2^)	18.4	99.9	56.5	23.6	0.62	30.7	383	68.2	32.0	0.49	**	17.1
TRV (cm^3^)	0.062	0.604	0.311	29.9	0.57	0.2	0.78	0.37	20.7	0.53	**	15.4
TRN	673.5	4804.5	1654.2	45.6	0.55	650	3620	1844	29.6	0.50	**	10.3
SFW (g)	1.291	4.889	3.243	20.1	0.72	0.68	3.35	1.4	17.5	0.47	**	− 131.8
SDW (g)	0.234	1.377	0.741	26.5	–	0.22	0.88	0.42	20.5	–	**	− 76.8
RFW (g)	0.261	0.788	0.503	19.2	0.60	0.27	1.62	0.58	18.9	0.50	**	13.4
RDW (g)	0.013	0.134	0.070	28.2	–	0.04	0.24	0.08	23.9	–	**	16.1
TFW (g)	1.617	5.599	3.746	19.3	0.70	1.02	4.98	1.98	16.6	0.58	**	− 89.2
TDW (g)	0.277	1.476	0.809	25.9	–	0.27	1.12	0.5	19.3	–	**	− 60.7
RSRF	0.102	0.248	0.159	15.9	0.70	0.27	0.7	0.43	14.5	0.63	**	62.9
RSRD	0.031	0.298	0.095	27.5	–	0.11	0.37	0.21	22.9	-	**	53.8

*CV* is coefficient of variation, *h*^2^ represent heritability, *ns* not significant

*P < 0.05

**P < 0.01, significance based on the analysis of variance

**Fig. 1:**
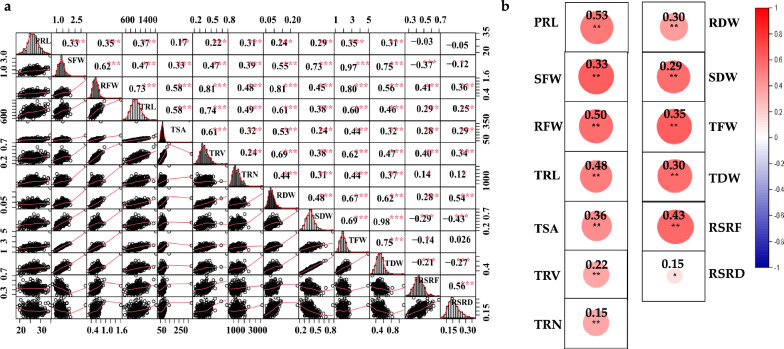
Correlation analysis of the investigated traits. **a** Correlations of studied traits under low phosphorus stress. The frequency distribution for each trait was displayed on the diagonal. The upper and lower parts represent the correlation coefficient and scatter plots between two diagonal traits, respectively. **b** Correlations of each investigated trait between control and low phosphorus stress. Red and blue indicate positive and negative correlations, respectively. ***, ** and * denote significance at the 0.1%, 1% and 5% levels of probability, respectively

The Pearson’s correlation coefficients between the measured traits studied under LP and CK treatments are presented in Fig. 1a–b. Obviously, SFW was found to be positively and significantly correlated with RFW under LP stress (*r* = 0.62, *P* < 0.01), which was consistent with the correlation under the control condition [28]. Root morphology contributed significantly to the formation of biomass-related traits, as demonstrated by the significant and strong correlation between SFW and TRL (*r* = 0.47, *P* < 0.01), TSA (*r* = 0.33, *P* < 0.01), TRV (*r* = 0.47, *P* < 0.01) and TRN (*r* = 0.39, P < 0.01). Moreover, negative correlations were also detected with the values of -0.37 between SFW and RSRF and -0.43 between SDW and RSRD, respectively (Fig. 1a). In addition, the Pearson correlation for root and biomass traits between control and LP-stress treatments showed the highest correlation for PRL (*r* = 0.53, *P* < 0.01) followed by RFW (*r* = 0.50, *P* < 0.01) and TRL (*r* = 0.48, *P* < 0.01). At the same time, TRN exhibited the lowest correlation (*r* = 0.15, *P* < 0.01) (Fig. 1b). Further, the study illustrated that multiple traits interrelated to PUE should be considered for a comprehensive assessment of RSA traits. Furthermore, normal and continuous frequency distributions were observed under LP stress for the studied traits, indicating that the investigated accessions would be appropriate for subsequent association studies (Fig. 1a).

### Marker–trait association analysis for root and biomass traits under LP-stress

**Fig. 2:**
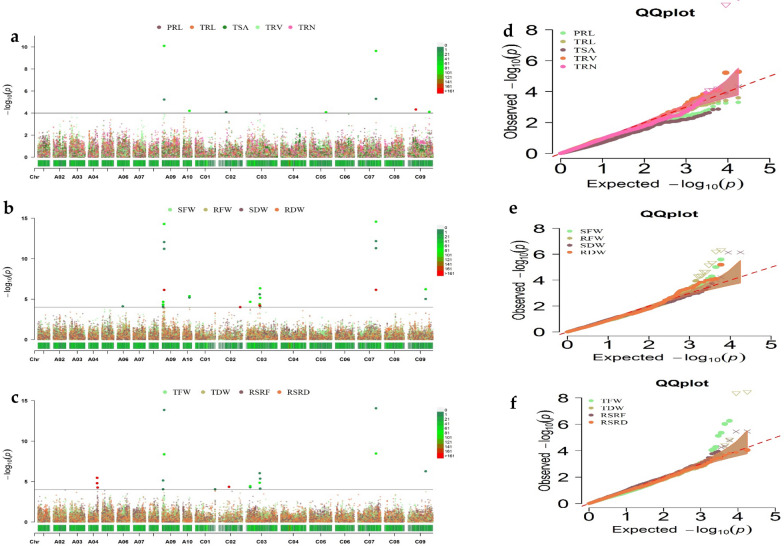
**a**–**c** Manhattan plots of the phenotype–genotype association analysis for 13 root and shoot biomass traits of B. napus by MLM with BLUE values. The x-axis displays the chromosome label, and the y-axis displays −log10 (p-value). The solid gray line shows significant associations between SNPs and phenotype value with a threshold level of the p-value (−log10 1/20, 131 = 4.30 × 10−5). The colour dots above the threshold values indicate the significant SNPs for root and shoot biomass traits. **d**–**f** QQ plots represent MLM analysis of the investigated traits

*Brassica 50 K Illumina Infinium* SNP array containing 45,708 SNPs were used for genotyping the association panel. Following SNP filtering, 20, 131 SNP markers were further used to find an association between traits and SNPs [28]. Since the results of association analysis under normal conditions were already reported [29], this study only conducted GWAS with BLUE values from three trials under LP stress (Fig. 2a–f). SNPs with close proximity (within 1 MB) and an LD r2 > 0.2 were considered as one QTL since these SNPs are tightly linked [41].

Thirty-nine marker–trait associations SNPs were found with a significant threshold of − log_10_ *P*-value > 4.30, which was distributed over seven linkage groups, varying from 1 on chromosome C02 to 12 on chromosome A09 (Additional file 2: Table S1). The important SNPs related to examined traits were displayed on Manhattan and QQ plots (Fig. 2a–f). Due to the complex nature of RSA traits and the strict MLM criteria, we determined suggestive trait–SNP associations (3.50 < log10 P ≤ 4.30) [29]. As a result, 39 significant SNP associations, with 15 significant SNP markers and 31 suggestive trait–SNP associated, were integrated into 11 valid QTL clusters (Tables 3, Additional file 2: Table S1), which included at least two investigated root and biomass-related traits. These SNP markers were detected except PRL, TRL, and RSRD for ten investigated traits. These QTLs were distributed on seven chromosomes; A04, A09, A10, C02, C03, C07, and C09. The highest number of loci were identified on A09 and C03, containing 28 and 19 loci (Additional file 2: Table S1).

**Table 3:** List of important QTL clusters for investigated traits under LN-stress in association panel

SNP cluster	No. of SNPs	Peak SNP	LG	Position	PVE (%)	*P*-Value	Haplotype block (Mb)	Traits
*RT-A04-1*	3	seq-new-rs23378	*A04*	17,194,300	8.62	5.45	16.88–17.19	RSRF
*RT-A09-1*	12	Bn-A09-p1552993	*A09*	2,375,212	8.15	5.13	2.22–3.42	TFW, SFW, RFW, RDW, TRV
*RT-A09-2*	16	seq-new-rs41996	*A09*	4,405,703	24.43	14.28	4.02–4.91	TDW, RFW, TFW, RDW, SFW, TRN, SDW, TRV
*RT-A10-1*	5	Bn-A10-p11396195	*A10*	12,709,829	8.61	5.37	12.68–12.72	RFW, RDW, TRN
*RT-C02-1*	1	seq-new-rs37919	*C02*	20,288,793	6.84	4.35	20.17–20.28	RSRF
*RT-C03-1*	5	seq-new-rs28219	*C03*	8,417,394	7.12	4.67	8.32–8.41	RFW, TDW, TFW, SDW, RDW
*RT-C03-2*	6	Bn-scaff_16925_1-p105130	*C03*	26,467,466	9.29	6.03	26.46–26.58	TFW, SFW, TDW, RFW, SDW, TRN
*RT-C03-3*	8	seq-new-rs41373	*C03*	27,421,341	9.88	6.33	27.42–27.46	RFW, TFW, SFW, RDW, TRV
*RT-C07-1*	8	seq-new-rs46512	*C07*	35,123,112	24.04	14.57	35.12–35.17	RFW, TFW, RDW, SFW, TRN, TDW, SDW, TRV
*RT-C09-1*	*1*	Bn-scaff_17888_1-p183402	*C09*	15,811,513	4.34	6.52	15.74–15.82	TSA
*RT-C09-1*	5	seq-new-rs34959	*C09*	34,541,392	9.75	6.26	34.38–34.54	TFW, RFW, SFW, TDW, TRV

Due to significant and strong relationships between root and biomass traits, numerous pleiotropic genetic loci were identified, particularly QTL clusters *RT-A09-1, RT-A09-2, RT-A10-1, RT-C03-2, RT-C03-3, RT-C07-1,* and *RT-C09-1*, which influenced both root formation and aboveground biomass. Two pleiotropic SNP markers (seq-new-rs41996 and seq-new-rs46512) in the QTL clusters *RT-A09-2* and *RT-C07-1* revealed high phenotypic variance of 24.43% and 24.04%, respectively, for RFW and were associated with both root morphology traits (RMT) and biomass traits (BM). These loci that simultaneously influence root and shoot biomass traits could be promising loci for marker-assisted breeding upon validation.

### Differential gene expression analysis between high/low phosphorus efficient groups and high/low phosphorus stress tolerance groups

**Fig. 3:**
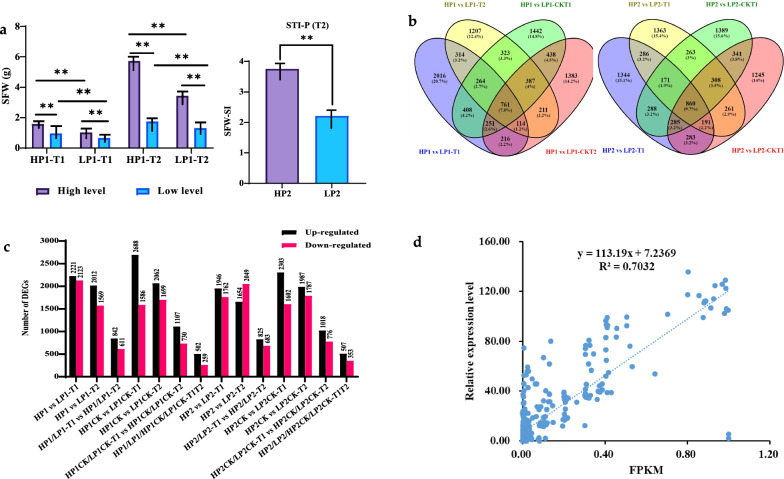
Differential gene expression analysis. **a** Phenotypic performance of high/low phosphorus efficient groups and high/low phosphorus stress tolerance groups. **b** Venn diagram of the DEGs in the selected groups. **c** Upand downregulated DEGs in different groups. **d** Correlation between qRT-PCR and RNA-seq data. ** and * denote significance at the 1% and 5% levels of probability, respectively. *ns*, not significant

Based on the marked differences in SFW at 7 (T1) and 14 (T2) days after transplanting, five accessions (S57, S178, S203, S233, S283) with higher SFW under both high/low P conditions were classified into the high phosphorus efficient (HP1) group. Five accessions (S55, S159, S202, S205, S269) with lower SFW under both high/low P conditions were classified as low phosphorus efficient (LP1). In addition, other ten accessions were evaluated based on the phosphorus stress tolerance index (STI, ratio of SFW under LP to control treatments) at 14 days and were classified into two groups; high STI (HP2, including S126, S178, S203, S219, S262) and low STI (LP2, including S33, S55, S59, S192, S311) (Fig. 3a). Therefore, 48 RNA-seq libraries were created, including three biological replicates of the HP1, LP1, HP2, and LP2 groups under low phosphorus stress and HP1CK, LP1CK, HP2CK, and LP2CK groups under control conditions at T1 and T2. The total, mapped, and unique reads to the reference *B. napus* genome are shown (Additional file 2; Table S2). After filtering the adaptor sequences and those with a low base quality, the Illumina sequencing produced 2280.3 million clean reads. At the same time, the average guanine-cytosine (GC) content was 47.4%, and the Phred quality score (Q30) was 88.8%. Using principal component analysis (PCA) and correlation analysis of gene expression levels, it was demonstrated that the correlation between individuals within the same group was much higher than the correlation between individuals within different groups (Additional file 1: Fig. S2a–b), indicating that the three biological repeats used were sufficiently accurate for the experiment.

A pairwise approach was used to determine the differentially expressed genes (DEGs) of the group-I (HP1 and LP1) under control and LP stress, respectively, including HP1 vs. LP1 and HP1CK vs. LP1CK under T1 and T2 points, respectively. Then the common DEGs of Group-I under both two-time points were found using a Venn diagram between HP1/LP1-T1 vs. HP1/LP1-T2 and HP1CK/LP1CK-T1 vs. HP1CK/LP1CK-T2. The same approach was used to screen the common DEGs of group II (HP2/LP2) that occur under both control and LP stress conditions at T1 and T2 time points (Fig. 3b). A false discovery rate (FDR) > 0.05 and a threshold of |log2 (fold change) |≥ 1, were used to determine the DEGs between these groups. Under the LP stress, 1453 common DEGs of group-I from the pairwise comparisons of HP1/LP1-T1 vs. HP1/LP1-T2 were identified, including 842 upregulated and 611 downregulated DEGs (Fig. 3c). Under the control condition, 1837 common DEGs of group-I from the pairwise comparisons of HP1CK/LP1CK-T1 vs. HP1CK/LP1CK-T2 were identified (1107 upregulated and 730 downregulated). Furthermore, the Venn diagram showed 761 DEGs (502 upregulated and 259 downregulated) overlapped between HP1 vs LP1-T1, HP1 vs LP1-T2, HP1 vs LP1-CKT1, HP1 vs LP1-CKT2 (Fig. 3b–c). This means that there were 692 DEGs specific to the HP1/LP1 group under the LP stress condition, while 1076 DEGs were specific under the control condition (Additional file 2: Table S3).

Similarly, 1508 common DEGs were identified from group II between HP2/LP2-T1 vs. HP2/LP2-T2 under the LP stress, including 825 upregulated and 683 downregulated DEGs and 1794 DEGs under the control condition (1018 upregulated and 776 downregulated) were identified between HP2CK/LP2CK-T1 vs. HP2CK/LP2CK-T2. Furthermore, 860 DEGs (507 upregulated and 353 downregulated) were regarded as common DEGs for HP2/LP2/HP2CK/LP2CK-T1/T2 (Fig. 3b–c). Considering this, HP2/LP2 showed 648 specific DEGs under the LP stress condition compared to 934 DEGs under the control condition (Additional file 2: Table S3).

DEGs' complete names, FPKM values, and accompanying descriptive information are presented in Additional file 2: Table S3. To categorize high/low P-specific DEGs, normalized FPKM values ranging from − 1 to 1 were employed to construct a heatmap based on expression profile similarity and diversity (Additional file 1: Fig. S3a–f). According to the heatmap, the expression patterns of DEGs were clearly shown as clusters of upregulated and downregulated genes. The qRT-PCR results for seven DEGs in all samples were strikingly similar to those from RNA-Seq, demonstrating the accuracy of the RNA-Seq data (Fig. 3d).

### Functional classification of DEGs involved in high/low phosphorus efficiency and high/low phosphorus stress tolerance index

To determine the functional significance of the DEGs in each group at two time points (7 and 14DAT), Gene Ontology (GO) and Kyoto Encyclopedia of Genes and Genomes (KEGG) classifications were identified for the DEGs in HP1/LP1-specific, HP1CK/LP1CK-specific, HP1/LP1/HP1CK/LP1CK-common, HP2/LP2-specific, HP2CK/LP2CK-specific, and HP2/LP2/HP2CK/LP2CK-common. Annotated genes were further divided into three major functional classifications; molecular functions (MF), cellular components (CC), and biological processes (BP).

**Fig. 4:**
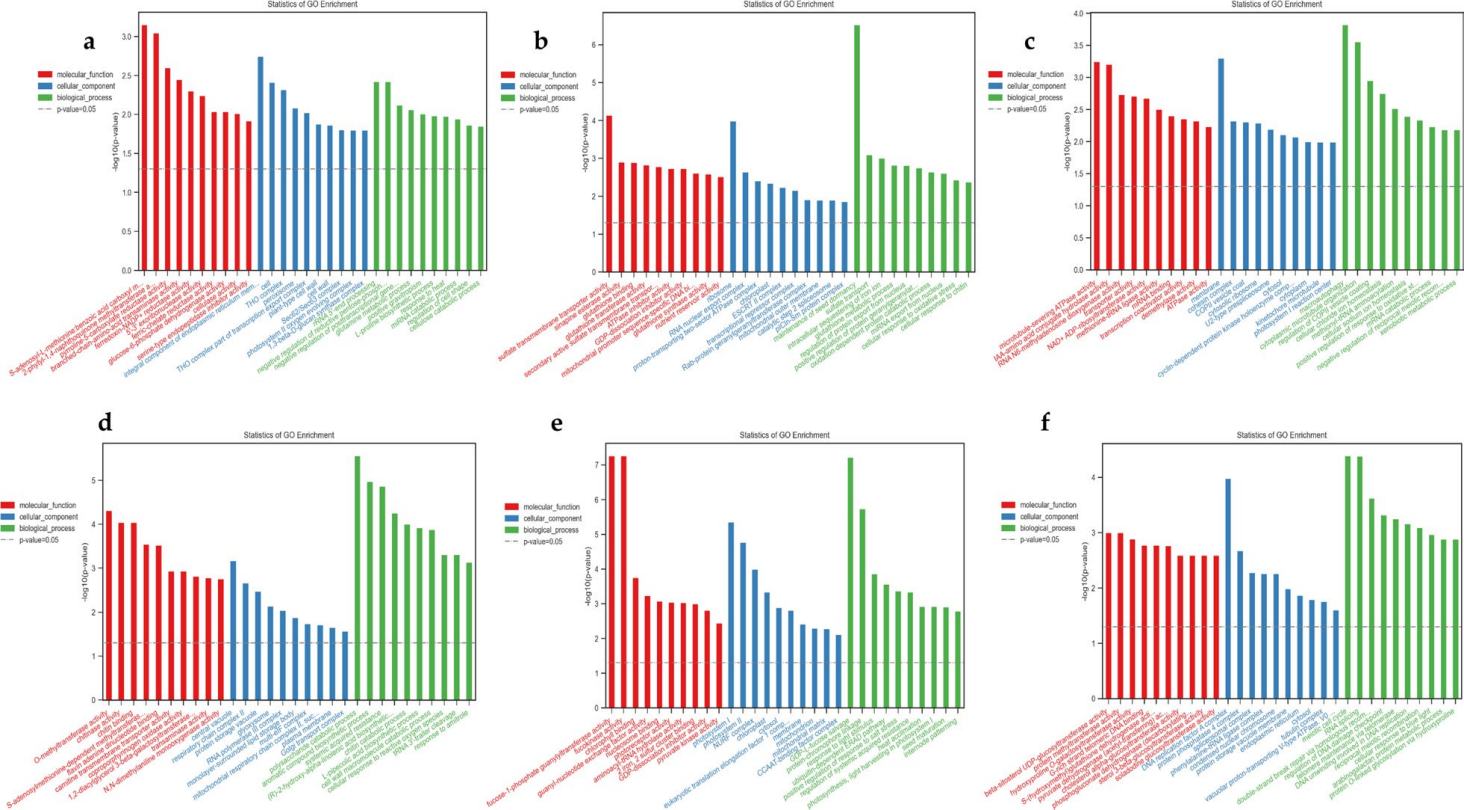
Gene ontology (GO) analysis of differentially expressed genes. **a**–**c** GO terms correspond to HP1/LP1-specific, HP1CK/LP1CK-specific, HP1/LP1/HP1CK/LP1CK-common, HP2/LP2-specific, HP2CK/LP2CK-specific, and HP2/LP2/HP2CK/LP2CK-common, respectively. **d**–**f** GO terms correspond to HP2/LP2-specific, HP2CK/LP2CK-specific, and HP2/LP2/HP2CK/LP2CK-common, respectively. The Y-axis is −log10 (p-value). The relevant p-value decreases as the bar chart height increases. Red, blue, and green colours correspond to molecular function, cellular components, and biological processes

Among the HP1/LP1 DEGs specific under LP stress, 543 (78.46%) of 692 received GO functional annotation. The most over-represented GO terms are displayed in Fig. 4a (according to their p-values). In the MF category, the most abundant GO terms were: benzoic acid carboxyl methyl transferase activity, methyltransferase activity, and carboxylate reductase activity. In the CC category, endoplasmic reticulum membrane and cell, followed by THO complex, were the most significant GO terms. Under the BP category, the DEGs were significantly enriched in; rRNA 5'-end processing, negative regulation of reductive pentose-phosphate cycle, and negative regulation of posttranscriptional gene silencing (Fig. 4a, Additional file 2: Table S4). Similarly, GO function annotations were obtained for 984 of the 1076 (91.44%) DEGs unique to HP1CK/LP1CK. Most transcripts enriched in the MF category were; sulfate transmembrane transporter activity, sinapine esterase activity, glutathione binding, and nutrient reservoir activity. Enrichment for DEGs associated with the CC category was; ribosome, RNA nuclear export complex, followed by proton-transporting two-sector ATPase complex. The BP category highly enriched the maintenance of seed dormancy, sulfate transport, intracellular iron ion sequestering, root meristem identity maintenance, and photosynthesis (Fig. 4b, Additional file 2: Table S4). The GO-term enrichment analysis for HP1/LP1/HP1CK/LP1CK-common DEGs also revealed important GO terms (Fig. 4c, Additional file 2: Table S4). The common GO terms were related to; microtubule-severing ATPase activity, transporter activity, phosphatide phosphatase activity, membrane, photosystem I reaction center, cellular chloride ion homeostasis, positive regulation of response to oxidative stress, photosynthesis, hormone-mediated signaling pathway, and carbohydrate transmembrane transport, sucrose synthase activity, activation of MAPK activity and glutathione metabolic process.

Similarly, GO enrichment analysis was performed for HP2 and LP2 at T1 and T2 under both LP stress and control treatments. A total of 648 HP2/LP2-specific, 934 HP2CK/LP2CK-specific, and 860 HP2/LP2/HP2CK/LP2CK-common DEGs were assigned to 203, 186, and 194 significant GO terms, respectively (Additional file 2: Table S4). For HP2/LP2 specific transcripts, the dominant terms were; methyltransferase activity, chitinase activity, L-glutamate transmembrane transporter activity, NADP binding, chloroplast, and RNA polymerase III complex, polysaccharide catabolic process, chitin catabolic process, cellular response to reactive oxygen species, regulation of phosphate transport, lateral root branching, and positive regulation of abscisic acid-activated signaling pathway (Fig. 4d, Additional file 2: Table S4). In group HP2CK/LP2CK-specific, the most enriched GO terms were; chlorophyll-binding, nutrient reservoir activity, photosystem I, photosystem II, photosynthesis, photosystem II assembly, and root hair cell tip growth (Fig. 4e, Additional file 2: Table S4). Moreover, 860 DEGs (Fig. 3b) that overlapped between HP2/LP2 and HP2CK/LP2CK were significantly enriched in different GO terms such as protein methyltransferase activity, NADP binding, proton-transporting ATPase activity, SUMO binding, DNA replication factor A complex, regulation of chlorophyll biosynthetic process, cellular response to glucose stimulus, cellular response to phosphate starvation, and carbohydrate metabolic process (Fig. 4f, Additional file 2: Table S4).

Based on the GO and KEGG results, sucrose synthase activity, DNA-binding transcription factor activity, auxin transport, lipid biosynthetic process, fatty acid degradation, phenylpropanoid biosynthesis, and tryptophan metabolism were unique pathways for low phosphorus uptake. In contrast, glutathione synthase activity, nutrient reservoir activity, sucrose-phosphate synthase activity, cellular response to oxidative stress, and glutathione metabolism were the important specific pathways for high phosphorus uptake. ATPase activity, phosphate binding, photosynthesis, response to oxidative stress, starch biosynthetic process, and selenocompound metabolism were common pathways for low/high phosphorus uptake. Furthermore, on the other hand, amino acid binding, electron transfer activity, NADP binding, lateral root branching, propanoate metabolism, and cellular response to sucrose starvation were the critical pathways for low/high phosphorus stress tolerance. In contrast, chlorophyll-binding, potassium ion binding, translation elongation factor activity, entrainment of the circadian clock, carbohydrate metabolic process, and amino sugar and nucleotide sugar metabolism were the most crucial pathways for low/high phosphors stress tolerance under control conditions. The most important pathways that were common in low/high phosphorus stress tolerance; transcription coactivator activity, SUMO binding, regulation of chlorophyll biosynthetic process, carbohydrate metabolic process, negative regulation of iron ion transport, cyanoamino acid metabolism, and glycerolipid metabolism. Therefore, it can be speculated that the genes associated with these pathways play an important role in regulating the low phosphorus responsiveness of rapeseed.

### Candidate genes prediction and prioritization by integrating GWAS, DEGs, and WGCNA

**Fig. 5:**
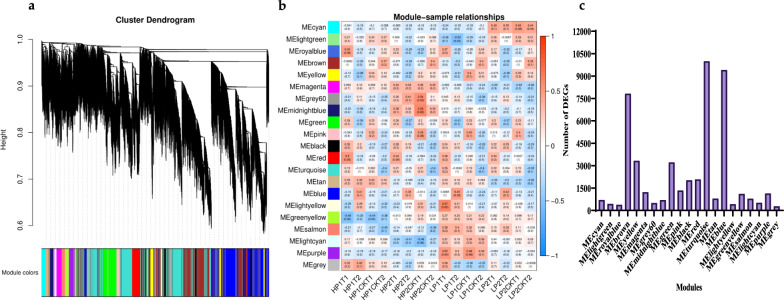
WGCNA of gene expression matrix. **a** Gene-based co-expression network analysis dendrogram. **b** Module–sample association; each row represents a module labelled with the same colour as in (**a**), and each column represents a sample. **c** Overview of identified genes corresponds to each module

An analysis of co-expression gene modules from 83,088 annotated genes with *p* > 0.05 was conducted to investigate the gene regulatory network under low phosphorus stress using WGCNA. In Fig. 5a, the dendrogram identified 21 modules based on gene correlation, and Fig. 5b depicts the relationships between modules and samples. A total of 48,454 genes were determined to be involved in these 21 modules, ranging from 292 in the ‘MEgrey’ module to 10,006 in the ‘MEturquoise’ module (Fig. 5c), indicating the complexity of gene regulation during phosphorus stress treatment. The MEtan, MEpurple, MEmidnightblue, and MEcyan modules were found to be highly correlated with HP1/CK-T1T2, LP1/CK-T1T2, HP2/CK-T1T2, and LP2/CK-T1T2, respectively (Fig. 5b). Based on the heatmaps, genes within one module were significantly expressed in samples that were strongly correlated with that module (Additional file 1; Fig. S4a–d).

The GO and KEGG analysis suggested that the significant GO terms of genes in the MEbrown module were related to glutamate-cysteine ligase activity, phosphatidate phosphatase activity, auxin binding, ATPase activator activity, response to ethylene, regulation of the salicylic acid metabolic process, cytokinin-activated signaling pathway, lateral root branching and formation and post-embryonic root development (Additional file 2: Table S5). Pyruvate metabolism, riboflavin metabolism, propanoate metabolism, N-Glycan biosynthesis, and inositol phosphate metabolism were significantly enriched in the “MEbrown” module by KEGG pathway enrichment analysis (Additional file 2: Table S6). These critical pathways were crucial in the phosphorus metabolism and assimilation process.

**Fig. 6:**
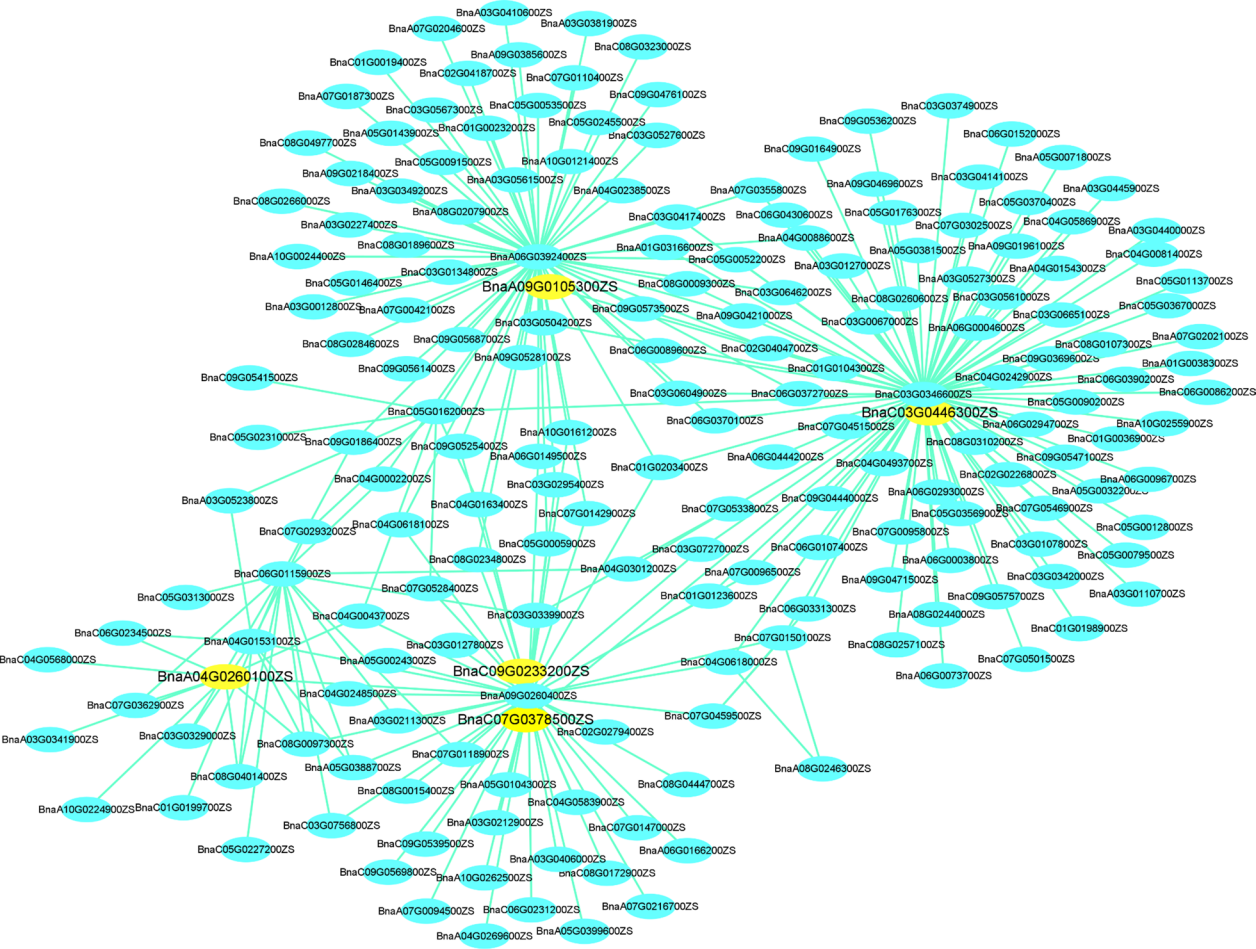
Correlation of networks in MEbrown module. The yellow colour in the network indicates the candidate genes overlapped by GWAS and WGCNA

The LD approach was used to identify candidate genes within 300 kb upstream and downstream of the significant peak SNPs associated with the traits studied [42, 43]. Based on GWAS results, 1060 genes were identified around each peak SNP in 11 QTL clusters located within the 300-kb region (Additional file 2: Table S7). We explored five potential genes that overlapped with GWAS and WGCNA genes based on the substantial and consistent correlation between WGCNA genes and each module (Table 4, Fig. 6). These five hub genes were found within MEbrown having high and consistent correlation across the samples under both phosphorus stress and control conditions. In addition, GWAS and DEG integration allowed us to identify seven genes simultaneously as common candidate genes. These candidate genes were detected within seven QTL clusters; *BnaA04g23490D* and *BnaA04g23610D* (*RT-A04-1*), *BnaA09g04320D*, *BnaA09g04350D* and *BnaA09g04930D* (*RT-A09-1*), *BnaA09g08440D*, *BnaA09g08940D* and *BnaA09g09290D* (RT-A09-2), *BnaC02g23620D* (*RT-C02-1*), *BnaC03g41900D* (*RT-C03-1*), *BnaC07g30970D* (*RT-C07-1*) and *BnaC09g18800D* (*RT-C09-1*). Within these 12 genes, six genes (*BnaA04g23490D, BnaA09g08440D, BnaA09g04320D, BnaA09g04350D, BnaA09g04930D, BnaA09g09290D*) that showed differential expression were identified previously related to phosphorus use efficiency, phosphorus utilization, assimilation, and root growth and development [44–46]. These findings evaluated the efficacy of an integrated GWAS, WGCNA, and differential expression analysis strategy for screening potential genes.

**Table 4:** List of candidate genes identified through integration of GWAS, WGCNA and differential expression analysis

Darmor_ID	HP1-T1	HP1-T2	HP1CK-T1	HP1CK-T2	LP1-T1	LP1-T2	LP1CK-T1	LP1CK-T2	HP2-T1	HP2-T2	HP2CK-T1	HP2CK-T2	LP2-T1	LP2-T2	LP2CK-T1	LP2CK-T2	QTL cluster	Distance from lead SNP (Kb)	Description
Hub genes in the MEbrown (GWAS + WGCNA)																			
BnaA04g23490D	29.04	16.93	36.05	70.89	17.85	13.13	25.81	72.68	27.42	15.17	23.84	80.26	33.70	10.92	28.13	76.21	RT-A04-1	− 249.41	Rhodanese-like domain-containing protein 7
BnaA09g08440D	3.00	2.21	2.95	5.04	3.31	1.81	2.27	4.24	2.67	1.57	2.26	4.52	2.30	2.11	2.70	5.41	RT-A09-2	249.992	BTB/POZ domain-containing protein NPY2
BnaC03g41900D	4.84	4.19	5.35	6.46	4.70	4.43	4.82	7.96	5.45	3.42	5.41	7.94	4.47	3.82	4.34	6.86	RT-C03-2	− 276.77	F-box/kelch-repeat protein At1g51550
BnaC07g30970D	14.99	17.71	17.37	11.43	16.22	20.68	19.31	11.02	18.75	19.21	19.33	10.65	16.34	20.22	18.62	11.28	RT-C07-1	− 118.79	Exocyst complex component EXO70E2
BnaC09g18800D	10.58	9.65	11.60	15.68	10.66	8.90	11.02	16.66	9.81	8.23	10.80	18.13	9.27	7.66	11.29	15.37	RT-C09-1	127.89	F-box protein SKIP31
Significant DEGs from GWAS + RNA-sequecing data																			
BnaA04g23610D	21.06	19.97	6.63	2.69	14.23	14.47	5.15	1.78	15.37	17.43	8.03	2.09	8.24	15.31	6.31	1.64	RT-A04-1	− 292.05	Nodulin MtN21 /EamA-like transporter family protein
BnaA09g04320D	0.32	0.11	1.80	4.13	0.64	0.29	1.94	4.61	0.62	0.24	1.50	6.33	0.73	0.11	1.40	5.81	RT-A09-1	248.43	ChaC-like family protein
BnaA09g04350D	2.34	0.57	9.49	16.89	0.90	1.27	5.38	20.24	1.51	0.97	6.22	17.87	1.90	0.56	3.07	15.99	RT-A09-1	238.58	Mitochondrial substrate carrier family protein
BnaA09g04930D	5.41	7.14	2.86	0.52	9.88	12.89	4.98	1.44	9.02	8.95	4.22	0.56	10.34	11.30	4.61	1.22	RT-A09-1	− 54.61	SALT OVERLY SENSITIVE 3 (SOS3)
BnaA09g08940D	0.80	0.52	2.72	2.50	0.72	0.56	2.92	1.40	0.83	1.00	2.45	1.65	0.66	0.24	2.04	2.15	RT-A09-2	− 41.72	NAC domain containing protein 36 (NAC036)
BnaA09g09290D	0.84	0.56	1.93	2.10	0.78	0.38	2.66	3.45	0.52	0.63	0.97	3.12	1.00	0.51	3.38	4.18	RT-A09-2	− 231.26	Winged-helix DNA-binding transcription factor family protein
BnaC02g23620D	34.34	17.21	151.15	91.50	8.26	8.04	88.39	82.32	20.59	10.28	110.35	99.37	16.67	11.49	59.72	93.75	RT-C02-1	− 257.99	Plant defensin 1.2 (PDF1.2)

## Discussion

### Phenotypic variation for root and biomass traits under phosphorus stress

Improving phosphorus stress tolerance is a key requirement for reducing the usage of phosphorus fertilizers and expanding rapeseed farming. Plants modify their root system in low-resource environments, particularly in low-nutrient and drought-prone areas [47]. Root architectural traits are highly variable among rapeseed genotypes under the LP-stress condition [48]. Due to the diverse genetic background and wide geographical distribution of the genotypes of the association panel, there was a significant amount of variation observed in the present study for root and shoot biomass traits. Under control and stress treatments, different RSA traits demonstrated large variability within the gene pool and may be exploited to increase root traits through breeding. The associated population's extensive diversity was shown by a significant coefficient of variation (> 10%) for all investigated root and shoot biomass traits (Table 2). Seedlings cultivated under LP conditions had lower SFW and TFW but higher RSRF, SDW, and RSRD than seedlings grown under control (CK) conditions, as previously reported [11, 49]. This may be attributed to the fact that plants tend to allocate a greater proportion of biomass to their roots. Longer TRL, TRV, TSA, and TRN indicate an increased ability to acquire more phosphorus from the nutrient solution. The broad-sense heritability of the studied traits was moderate to high (0.55–0.72) and (0.47–0.63) under control and LP conditions, respectively (Table 2). Furthermore, most traits had a normal distribution (Fig. 1a). This implies that these root and biomass traits were more genetically regulated and, thus, more amenable to genetic improvement in low-P conditions. Other research revealed similar findings [50].

The relationship between root and biomass traits indicates a balance between root and shoot organs and partitioning resources between above- and below-ground tissues [51]. Most traits studied under control and LP treatments showed a significant positive correlation (Fig. 1a–b). Interestingly, RFW and TRL were highly correlated under LP conditions since these factors influence the plant's absorption of nutrients and water [52]. Furthermore, a high correlation between shoot and root dry weights might be due to nutrients supplied from roots to shoots, as seen in rapeseed [53]. Significant and high correlations among root and biomass traits were reported in rice under low phosphorus soil conditions [7, 54].

### GWAS analysis identifies some important genomic regions

PUE is an important trait that determines yield potential in several crops, including rapeseed. Crops with enhanced PUE lead to higher growth for the same amount of phosphorus taken up at a time [55]. Several studies have explored the genetic basis and function of roots in rapeseed under low phosphorus, and several loci have been reported across various populations and environments [56, 57]. Based on 20,131 high-quality SNPs in a 327-rapeseed association panel, 70 SNPs (39 significant and 31 suggestive) were linked with root and shoot biomass parameters that were further integrated into 11 reliable QTL clusters (Table 3, Additional file 2: Table S1). The estimated phenotypic variation for the studied traits ranged from 4.34–24.43%, demonstrating that several loci influence RSA traits with small to moderate effects. This also explained the intricate genetic control of these traits at an early stage of crop development. The current study findings identified several loci which were in line with the previous reports on several dynamic traits in different crops [48, 58–61]. Seven QTL clusters (*RT-A901-1, RT-A09-2, RT-A10-1, RT-C03-2, RT-C03-3, RT-C07-1, RT-C09-2*) in the present study were co-localized with previously identified loci related to root and biomass traits under low nitrogen conditions using association mapping population [57], while two QTL clusters (*RT-A09-1, RT-C02-1*) were found co-localized with the previously identified loci (*qc.A09-2, qcC02-2*) related to root and biomass traits under low nitrogen, phosphorus, and potassium (NPK) and control conditions, respectively, using linkage mapping population in rapeseed [40, 49]. These findings indicate that these genomic regions may harbor genes with pleiotropic effects related to different root and biomass traits under low-phosphorus conditions at the rapeseed seedling stage. These results further confirm that these genomic regions may be highly valuable for identifying the genes associated with P-deficiency tolerance and PUE.

### Differential gene expression and weighted gene co-expression network analysis

Understanding the molecular mechanisms underpinning PUE is essential for optimizing crop yields with lower investments in phosphorus fertilizer. Over the past few years, RNA-Seq has been extensively used to investigate the transcriptome of a wide range of economically important crop plants under P deficit conditions [62, 63]. Comparative transcriptomics has been utilized successfully to investigate the fundamental genetic mechanisms in rapeseed [29, 57]. RNA-seq analysis was performed to investigate the molecular mechanisms involved in rapeseed seedling response to low phosphorus stress. The present study identified 692, 1076, 648, and 934 DEGs specific to HP1/LP1, HP1CK/LP1CK, HP2/LP2, and HP2CK/LP2CK, respectively, while 761 and 860 DEGs were found common for HP1/LP1/HP1CK/LP1CK and HP2/LP2/HP2CK/LP2CK groups, respectively (Fig. 3b–c, Additional file 2: Table S3). DEGs associated with significant GO terms might have a critical role in root growth and phosphorus efficiency/stress tolerance (Additional file 2: Table S4). For example, a gene *BnaC03G0388000ZS* (*ATBSMT1*) significantly associated with GO term benzoic acid carboxyl methyl transferase activity has been reported to regulate root growth and development under low phosphorus stress [64, 65]. *BnaA07G0336100ZS* (*ATPS2*), corresponding to the term phosphatase activity, has been documented that play an important role in phosphate starvation in *Arabidopsis thaliana* [66]. *BnaC09G0364500ZS* (*AtILL3*), related to IAA-amino acid conjugate hydrolase activity that encodes a superfamily protein, has been reported to regulate root morphogenesis at the seedling stages [67]. *BnaA07G0131000ZS* (*AtATL80*), associated with regulating phosphate transport that encodes RING E3 ubiquitin ligase, has been reported to play a crucial role in phosphorus stress signaling [68]. *BnaA03G0165600ZS* (*AtLHB1B1*), associated with the GO term ‘chlorophyll binding’, regulates root development and phosphorus use efficiency [5].

The integration of GWAS, WGCNA, and differential gene expression analysis is a promising strategy and has been utilized in many crops to explore potential candidate genes [57]. We identified five hub genes by integrating GWAS and WGCNA data. In addition, seven DEGs were determined by combining GWAS and differential gene expression data (Table 4). Several candidate genes were found that play a central role in root growth and development in response to phosphorus stress. For example, *BnaA04g23490D* (*AtSTR7*), a major hub gene located in the region of QTL cluster *RT-A04-1,* encodes phosphatase superfamily protein and is involved in primary root elongation in *A.thaliana* [44]. *BnaA09g08440D* (*ATNPY2*), another hub gene in the QTL cluster *RT-A09-2*, has been involved in root growth and development [57]. Four potential candidate genes out of seven were identified through the integration of GWAS and differential gene expression analysis that has been reported to function as central regulators in root development and phosphorus stress. *BnaA09g04320D* (*AtGGCT2;1*), a member of the *GAMMA-GLUTAMYL CYCLOTRANSFERASE family,* has been reported to regulate RSA under different abiotic stresses [69]. *BnaA09g04350D* (*AtPP4*) detected in RT-A09-1 is involved in root meristem activity during phosphorus starvation [70]. *BnaA09g04930D* (*AtCBL4*) has been found that play a central role in root meristem growth and development in response to various environmental cues [71]. *BnaA09g09290D* (*AtLARP1/RSL4*), a member of the DNA-binding transcription factor family protein, has been documented to regulate root hair elongation in P-limited conditions [72].

## Conclusions

Identifying regions on the chromosome in the form of QTLs to explain the phenotypic variation for the various breeding traits is an important tool to improve breeding program efficiency. The identified genomic loci in the present study explain the significant variation in phosphorus uptake and utilization or its associated root and biomass traits. The discovered common QTLs controlling several phenotypes may serve as candidate markers for marker-assisted breeding. Given phosphorus's rising cost and relevance as an agricultural input, Crop improvement, PUE is an intrinsically worthwhile objective. However, a PUE-focused breeding program will compete with other breeding goals like disease resistance and climate change adaptability. The discovery of QTL allows for the creation of trait-relevant markers for marker-assisted or genomic selection methods. Overall, the key loci/genes and metabolic pathways uncovered here may serve as valuable genetic resources that could be targeted for breeding phosphorus-efficient cultivars of rapeseed.

## Materials and methods

### Plant materials and growth conditions

There were 327 rapeseed germplasm accessions used in this study, including 222 from the Yangtze River, 52 from other places/unknown origins, 23 from northwestern China, 16 from Europe, and 14 from Australia. These included 191 semi-winter accessions (P1), 34 winter accessions (P2), and 102 spring accessions (P3) [28]. All the accessions were strictly self-crossed.

A pilot experiment was conducted using eight B. napus lines from the association panel, comprised of H1, H4, H5, and H6 (semiwinter accessions), H2 and H3 (winter accessions), H7 and H8 (spring accessions). A completely randomized design was used to evaluate these eight lines at the Oil Crops Research Institute, Chinese Academy of Agricultural Sciences, Wuhan, China, using the hydroponic setup described previously [74]. According to Hoagland's solution, eight phosphorus concentrations (1, 0.1, 0.05, 0.025, 0.01, 0.007, 0.005, and 0.003 mM P+) were used with a consistent concentration of other elements [74]. Seeds were kept in darkness for two days on a medical gauze, then exposed to light (180 mol photons m^-2^s^-1^) and grown for 4 days under 16/8 hours of day/night cycles at 24 °C in a greenhouse (60–80% relative humidity). The full-strength modified Hoagland’s solution was composed of 5mM Ca (NO3)2·4H_2_O, 5mM KNO_3_, 2mM MgSO_4_ ·7H_2_O, 1mM KH_2_PO_4_, 0.05mM EDTA-Fe, 46 µM H3BO3, 9.14 µM MnCl_2_·4H_2_O, 0.77 µM ZnSO_4_·7H_2_O, 0.37µM NaMoO_4_·2H_2_O , and 0.32 µM CuSO_4_·5H_2_O. A quarter of Hoagland's solution was added to the seedlings' growth device six days after planting. A total of 24 seedlings of four different lines were planted in each basin (six seedlings for each line). Every week, nutrient solutions were changed. In the weeks following harvesting, the 1/4 solution was changed to a 1/2 solution, then to a 100% solution

The pilot experiment results were used for LP treatment of the association mapping population, which were evaluated using three independent trials with a CRD. With the consistent concentration of other elements, two *P* concentrations (1 mM L^−1^, control) and 0.01 mM L^−1^, stress) were applied in a hydroponic setup as described above.

### Phenotypic investigation

Three plants from each genotype were collected during harvest and divided into root and shoot sections. Five root morphology traits (RMT) viz. total root length (TRL), total root surface area (TSA), total root volume (TRV), and total root number (TRN) were captured through images using a scanner (EPSON V700, Japan) and further analyzed by WinRHIZO software (Pro 2012b, Canada), while primary root length (PRL) was measured manually using a ruler. Eight biomass-related traits (BT), including root fresh weight (RFW), shoot fresh weight (SFW), were measured manually by using a weighing balance. Root dry weight (RDW) and shoot dry weight (SDW) were measured after oven drying at 80 °C until a consistent weight was reached. Total dry weight (TDW) and total fresh weight (TFW) were estimated as SDW + RDW and SFW + RFW, respectively. The ratio of root-to-shoot fresh weight (RSRF) and the ratio of root-to-shoot dry weight (RSRD) was measured as the ratio between RFW and SFW and the ratio between RDW and SDW, respectively (Table 1).

### Statistical analysis

Statistical analysis was performed across three trials employing best linear unbiased estimate (BLUE) values for 13 traits investigated under phosphorus stress. With a significance threshold of 0.05, paired samples t-tests were used to assess statistically significant differences between treatments. Using QTL Ici mapping 4.2, basic statistics and broad-sense heritability were calculated [75]. With the ‘‘*PerformanceAnalytics*’’ package in R software, Pearson correlation was calculated at a significance level of (*P* < 0.05). The response of each trait to LP was represented by the increase or decrease of LP relative to CK, calculated as (LP-CK)/LP × 100%.

### Association analysis

By utilizing the BLUE values from three LP trials, a mixed linear model (MLM) with a (Q + K) matrix was used to investigate trait–SNP associations via Tassel 5.0 software [76]. The marker–trait association was determined by an arbitrary cutoff value of 1/20,131 SNPs (− log 10 (*p*) = 4.30). The qqman and ggplot2 packages were used to generate Manhattan plots and Quantile–Quantile plots, respectively [77, 78]. With the help of *Haploview* software, marker haplotypes at each linked locus were identified based on the four-gamete criterion [79].

### Candidate gene prediction

Based on *B. napus* '*Darmor*' reference genome information, a complete gene list was scanned in the QTL cluster region [80]. Based on Gene Ontology (GO terms) from the *TAIR* website and gene functions from prior studies, potential phosphorus efficiency/LP tolerance genes were identified [80].

### Transcriptome sequencing and analysis

Based on the differences in SFW, ten germplasms with extremely high or low SFW (high/low phosphorus efficient) and ten germplasms with extremely high or low P utilization index (the ratio of SFW under P stress and CK treatment) from the association population were selected. Roots of these accessions were collected alone under control and LP stress at two-time points, 7 and 14 days after transplanting (T1 and T2). Samples equally mixed into four groups, named group HP1 with high SFW (S57, S178, S203, S233, S283); group LP1 with low SFW (S55, S159, S202, S205, S269); group HP2 with high P utilization index: S126, S178, S203, S219, S262; group LP2 with low P utilization index: S33, S55, S59, S192, S311, respectively.

Each sample was analyzed for RNA-sequencing using three biological replicates from three independent plants. Forty-eight RNA-seq libraries (one-tissue × four groups × two treatments × two-time points × three biological replicates per group) were prepared for total RNA extraction with *IRIzol reagent* (Invitrogen, USA). An *Illumina HiseqTM 2500* platform was used by *OEBiotech* Company in Shanghai-China, to construct sequencing libraries and conduct Illumina sequencing. Raw readings with 150 paired-end base pairs (bp) were filtered and aligned [81].

A reference genome for *B. napus ZS11* and *HISAT v2.0.4* were used for mapping the clean reads (https://www.genoscope.cns.fr/brassicanapus/data/) [80]. The WGCNA was conducted using the WGCNA package in R [82]. The “DESeq” R package was utilized to identify DEGs using ≤ 0.05 for the false discovery rate (FDR) and |log2 ratio|≥ 1 as criteria.

### Validation of DEGs by quantitative real-time polymerase chain reaction (qRT-PCR)

As previously described, seven differentially expressed candidate genes were assessed by qRT-PCR to measure the reliability of the RNA-seq data [83]. The primer sequences are presented in Additional file 2: Table S8. The SYBR qPCR Master Mix (*Vazyme*) was used with the *CFX96* for qRT-PCR analysis (*BIO-RAD*). Each sample was subjected to three technical replications. The 2^−ΔΔCT^ method was utilized to determine the relative expression of target genes using *B. napus ACTIN2* as an internal control [84].

### Supplementary Information

**Additional file 1: Figure S1.** **a** SFW of the eight-rapeseed lines across the eight different P concentrations. **b** The ratio of SFW between the stress treatment and the control condition. **Figure S2.** **a** Principal component analysis (PCA) among the RNA-sequencing samples. **b** Pearson correlation coefficient among the RNA-sequencing samples. **Figure S3.** **a**–**f** Heat maps of HP1/LP1-T1T2, HP1CK/LP1CK-T1T2, HP1/LP1/HP1CK/LP1CK-T1T2, HP2/LP2-T1T2, HP2CK/LP2CK-T1T2 and HP2/LP2/HP2CK/LP2CK-T1T2, respectively. **Figure S4.** **a**–**d** Heat maps of the expression profiles of eigengenes in the MEtan, Empurple, MEmidnightblue, and MEcyan modules, respectively.

**Additional file 2: Table S1.** List of significant and suggestive SNPs for the investigated traits under LP-stress in the association panel. **Table S2.** Summary of read numbers and mapped reads from the RNA-Seq. **Table S3.** Information of DEGs (HP1/LP1-specific, HP1CK/LP1CK-specific, HP1/LP1/HP1CK/LP1CK-common, HP2/LP2-specific, HP2CK/LP2CK-specific, HP2/LP2/HP2CK/LP2CK-common). **Table S4.** Gene Ontology (GO) analysis for HP1/LP1-specific, HP1CK/LP1CK-specific, HP1/LP1/HP1CK/LP1CK-common, HP2/LP2-specific, HP2CK/LP2CK-specific and HP2/LP2/HP2CK/LP2CK-common, respectively. **Table S5.** List of significant GO terms in the MEbrown module. **Table S6.** List of significant KEGG pathways in the MEbrown module. **Table S7.** List of potential candidate genes within 300 kb upstream and downstream of the peak SNP in 11 QTL clusters for investigated traits under the LP stress. **Table S8.** Primers for qRT-PCR used in this study.

## Data Availability

The raw sequence data have been deposited in the National Center for Biotechnology Information Sequence Read Archive (http://www.ncbi.nlm.nih.gov/sra/) under Accession number PRJNA714285. The manuscript and additional files include all other relevant data during this study.
