# Supplementary figures and images for: Integrating genome-wide association study with transcriptomic data to predict candidate genes influencing Brassica napus root and biomass-related traits under low phosphorus conditions
Source: Biotechnol Biofuels Bioprod. 2023 Oct 4;16:149. doi: 10.1186/s13068-023-02403-2 (PMC10548562; doi:10.1186/s13068-023-02403-2)

## Slide 1
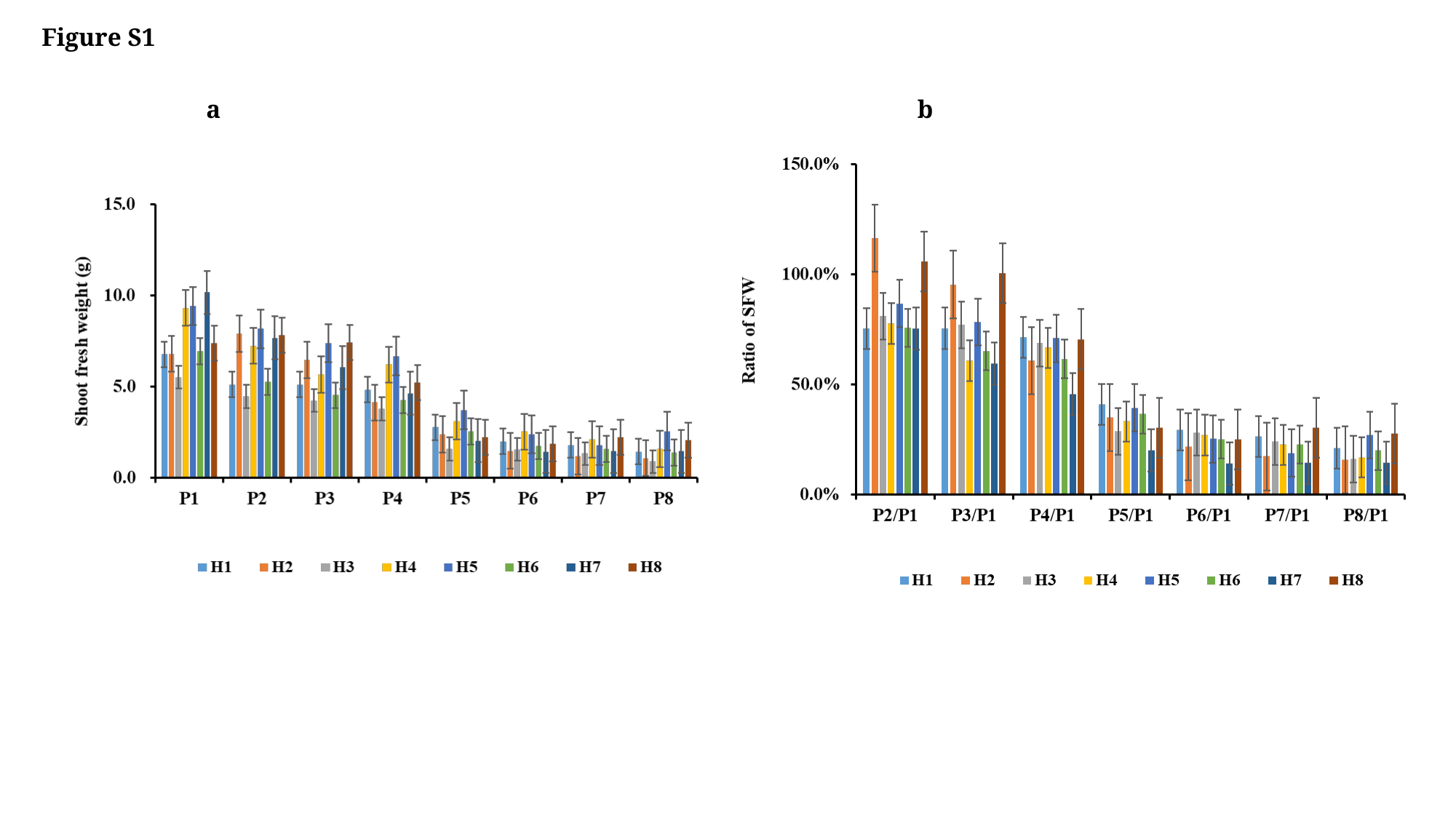

Figure S1
a
b

## Slide 2
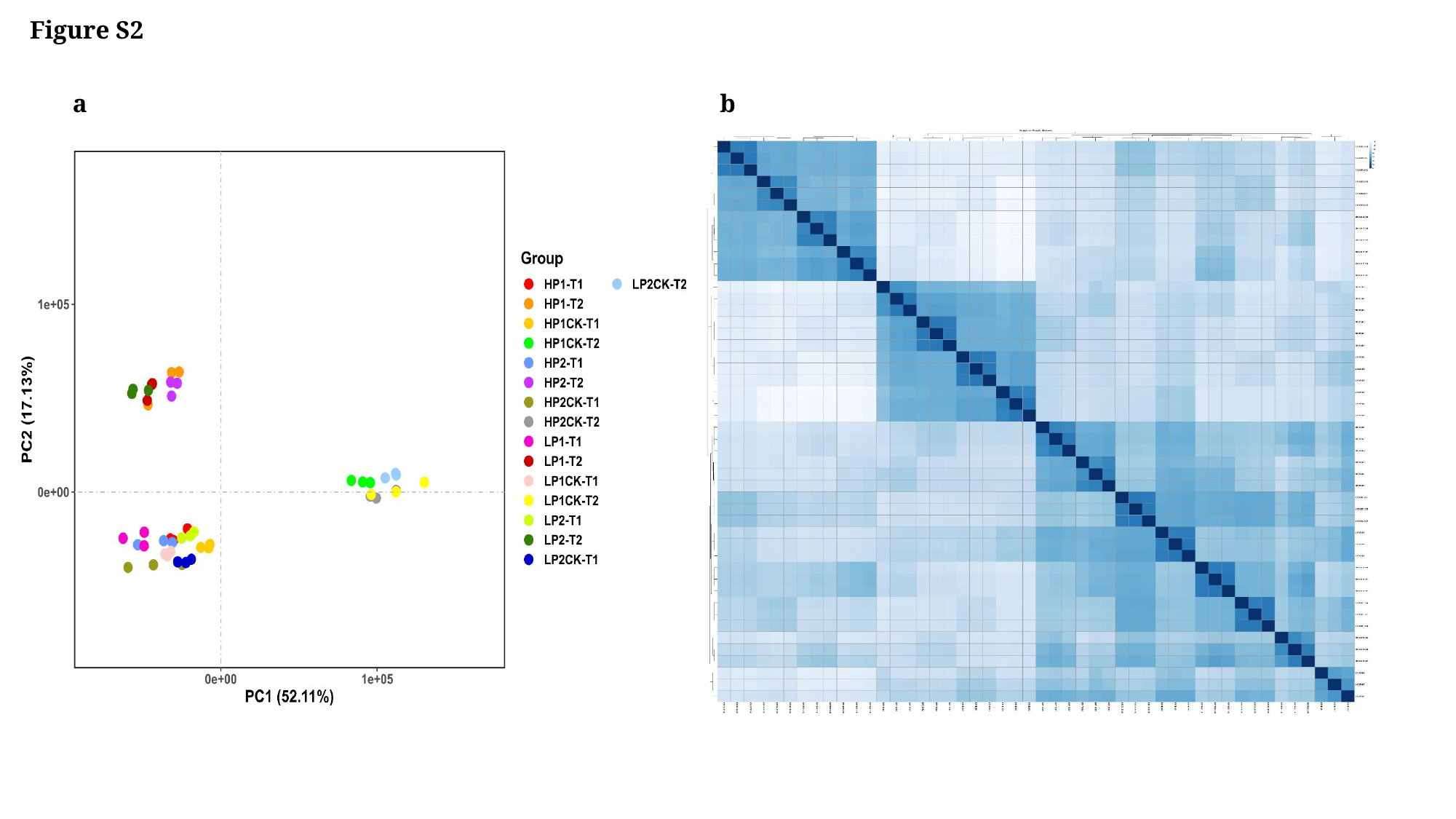

Figure S2
a
b

## Slide 3
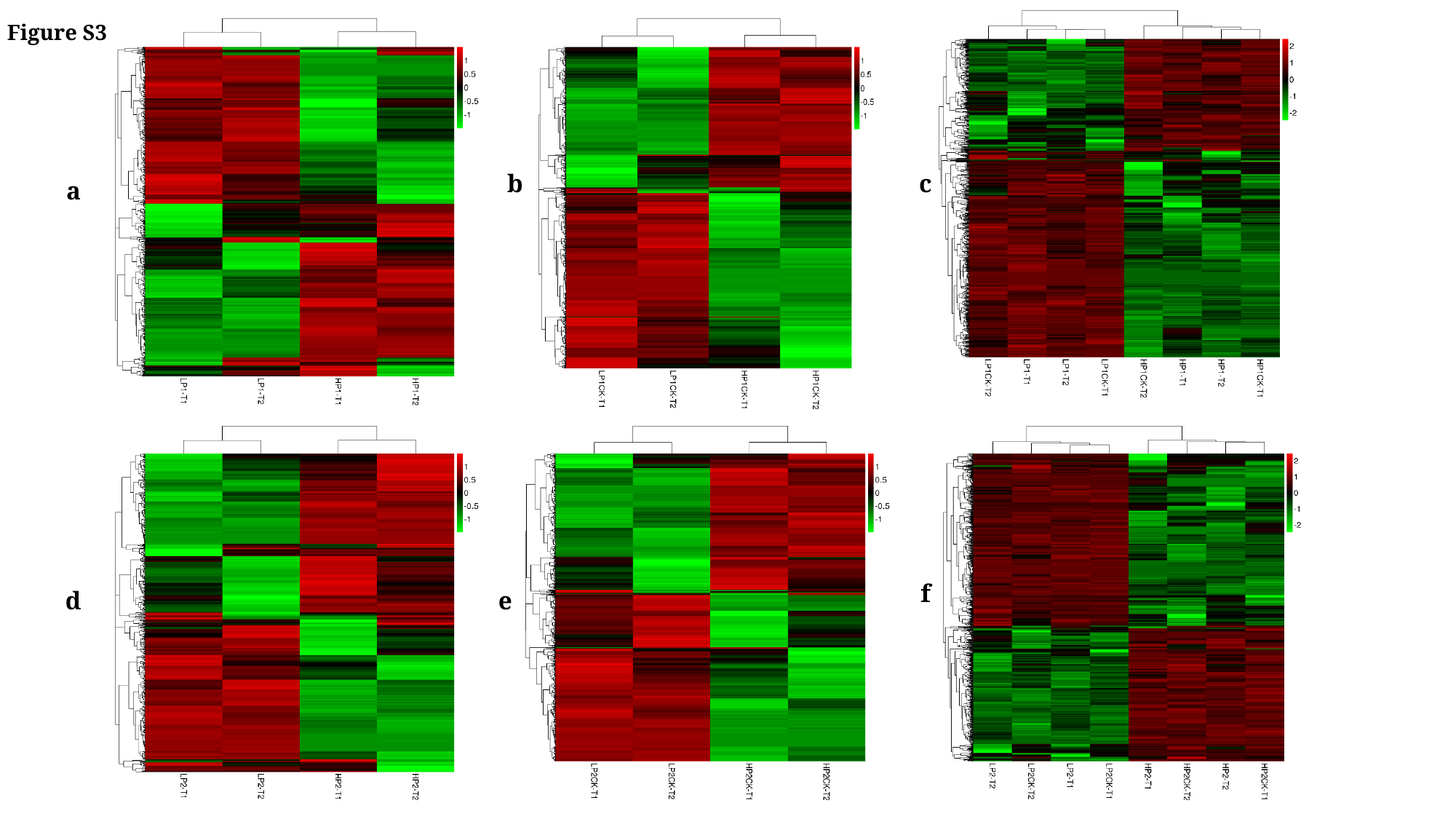

Figure S3
b
c
a
f
d
e

## Slide 4
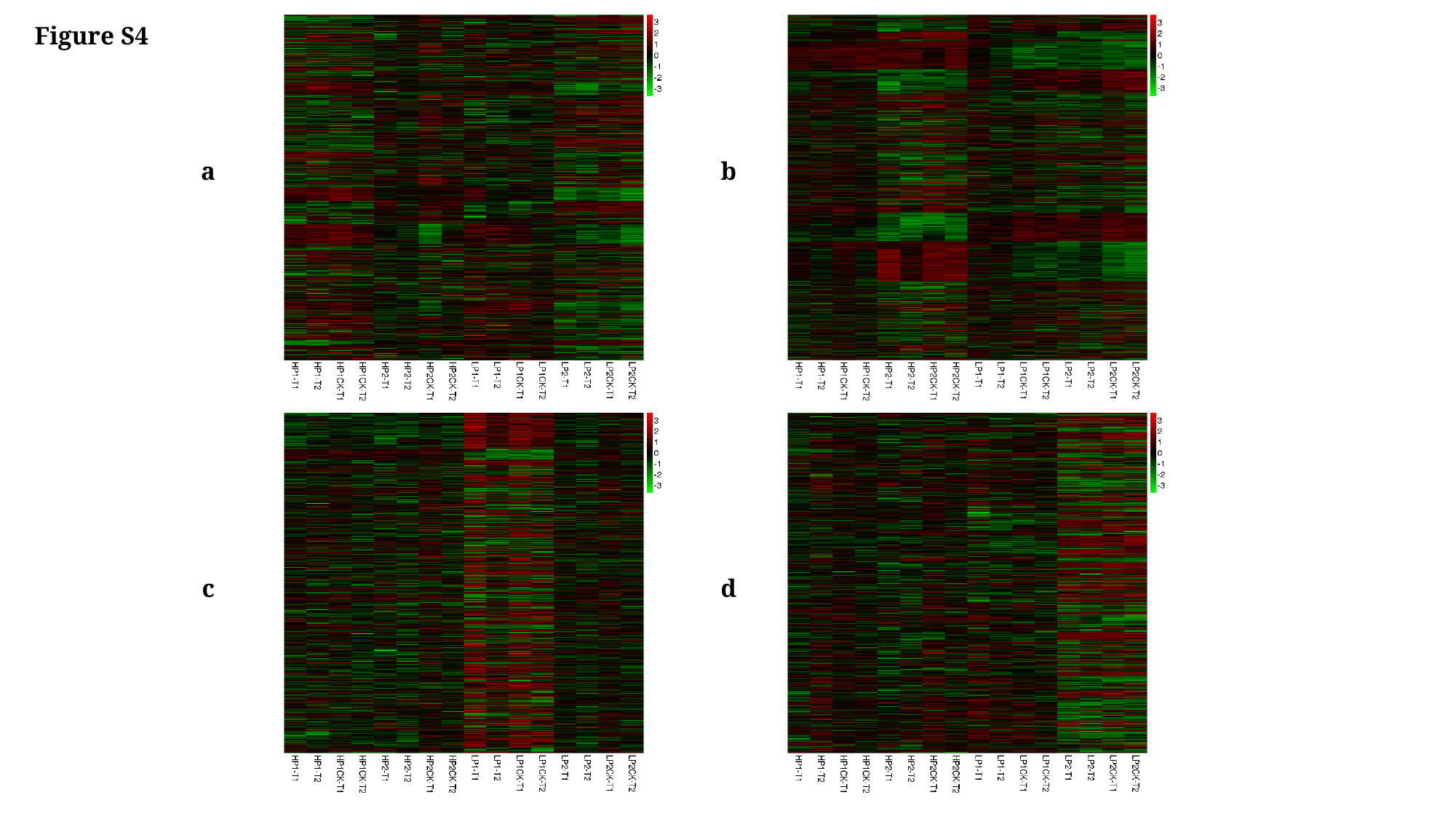

Figure S4
a
b
c
d

Supplement: Supplementary file 1 — Additional file 1: Figure S1. a SFW of the eight-rapeseed lines across the eight different P concentrations. b The ratio of SFW between the stress treatment and the control condition. Figure S2. a Principal component analysis (PCA) among the RNA-sequencing samples. b Pearson correlation coefficient among the RNA-sequencing samples. Figure S3. a–f Heat maps of HP1/LP1-T1T2, HP1CK/LP1CK-T1T2, HP1/LP1/HP1CK/LP1CK-T1T2, HP2/LP2-T1T2, HP2CK/LP2CK-T1T2 and HP2/LP2/HP2CK/LP2CK-T1T2, respectively. Figure S4. a–d Heat maps of the expression profiles of eigengenes in the MEtan, Empurple, MEmidnightblue, and MEcyan modules, respectively. [file 13068_2023_2403_MOESM1_ESM.ppt]
